# Phenotypic and Metabolic Variations Induced by Autopolyploidization in Chinese Jujube Cultivars

**DOI:** 10.3390/plants14233588

**Published:** 2025-11-25

**Authors:** Yan Han, Jing Sui, Tong Yao, Shuting Chen, Meng Yang, Miao He, Tingting Ye, Xiaoshan Li, Taoliang Song, Mengjun Liu, Ping Liu

**Affiliations:** 1 College of Horticulture, Hebei Agriculture University, Baoding 071001, China; hanyan5156340@126.com (Y.H.); 15614418002@163.com (J.S.); iamrigena67@126.com (T.Y.); 19811834582@163.com (S.C.); biomichael@163.com (M.Y.); maxine940412@outlook.com (M.H.); wsyetingting@126.com (T.Y.); lxs15032238094@163.com (X.L.); 2 Research Center of Chinese Jujube, Hebei Agricultural University, Baoding 071001, China; 3 Shijiazhuang Institute of Pomology, Hebei Academy of Agriculture and Forestry Sciences, Shijiazhuang 050061, China; taoliang918@126.com

**Keywords:** autotetraploid, morphology, metabolites, phytohormone, *Ziziphus jujuba Mill*

## Abstract

Autopolyploidization is critical to plant evolution and breeding, but systematic studies on its effects in woody plants remain limited. To address this gap, a systematic investigation covering autopolyploidization-induced variations in two autotetraploid jujube cultivars and their diploid counterparts across morphological, cytological, and metabolic levels was conducted in the present study. Compared with the diploids, the autotetraploid jujubes exhibited larger leaves, flowers, and fruits, but a dwarfing phenotype with reduced fruit set. Additionally, decreased leaf stomatal density and weakened pollen viability were observed in the autotetraploid jujubes. Metabolomic analysis further revealed that autotetraploid fruits accumulated higher contents of soluble sugars, flavonoids, phenolics, and alkaloids but contained lower contents of amino acids. Based on LC-MS/MS quantification of leaf phytohormones, we identified six common hormones that were differentially accumulated in both cultivar comparisons. Notably, all six showed consistent alteration patterns between the two autotetraploid–diploid pairs. Together, these findings enhance our understanding of how autopolyploidy influences secondary metabolism, plant architecture, and hormone homeostasis in woody plants.

## 1. Introduction

Autopolyploidization is more common in plants than traditionally assumed, but has received little attention compared with allopolyploidization [1]. Autopolyploids and allopolyploids are fundamentally different. Autopolyploidy, caused by chromosome duplication, differs from allopolyploidy, which involves both hybridization and chromosome doubling. Due to their less complex genetic makeup, autopolyploids thus serve as a good system for investigating how genome duplication influences gene expression [2]. Currently, evidence is limited but points to a clear finding: in the first few generations after genome doubling, autopolyploids undergo very little strong or extensive genetic recombination [3,4], However, over a longer period, these recombination processes may become more important [5,6]. Christian Parisod et al. hypothesize that polysomic inheritance may provide a short-term evolutionary advantage for autopolyploids compared to their diploid relatives when environmental change enforces range shifts. In addition, autopolyploids should possess increased genome flexibility, which allows them to adapt and persist across heterogeneous landscapes in the long run [1].

Although whole-genome duplication does not cause large-scale genomic restructuration and DNA changes, it significantly influences plant morphological traits, metabolite content, phytohormone levels, as well as gene expression and regulation [7].

Autopolyploid plants typically exhibit a suite of characteristic morphological and cytological features, including larger organs, reduced stomatal density, dwarfism, and the production of larger but less viable pollen grains [8,9,10,11,12,13]. Beyond these structural changes, autopolyploidization profoundly influences secondary metabolism, often enhancing both the quantity and quality of metabolites. For instance, studies on *Thymus persicus* (triterpenoids) [14] and *Tetradenia riparia* (essential oils) [15] report quantitative increases, while research on alkaloid-producing species reveals more complex shifts. In *Datura stramonium*, genome duplication not only increases total alkaloid content but also alters the scopolamine/hyoscyamine ratio [16]. A more dramatic change is observed in *Hyoscyamus reticulatus*, where autotetraploidy drastically upregulates the conversion of hyoscyamine to scopolamine, elevating its content from 0.23% in diploids to 8.66% in tetraploids [17].

Polyploidization also reshapes the plant’s endocrine profile. In autopolyploid *Artemisia annua*, it enhances not only photosynthetic capacity but also the endogenous levels of indole-3-acetic acid (IAA), abscisic acid (ABA), and jasmonic acid (JA), alongside increases oxidative stress [18]. The role of hormones in mediating polyploid traits is further illustrated by a study on cold resistance in jujube. The diploid cultivar ‘Yueguang’ demonstrates superior cold tolerance over its autotetraploid counterpart ‘Hongguang’, as indicated by a higher xylem-to-cortex ratio, lower semi-lethal temperature, and reduced malondialdehyde content. Transcriptomic and exogenous hormone analyses suggest that ABA and brassinolide (BR) are crucial in regulating this differential cold resistance [19].

At the molecular level, polyploidization exerts broad effects on gene expression and epigenetic regulation. Gao et al. reports that tetraploid citrus exhibits lower CG methylation levels in gene and transposable element (TE) bodies compared to diploids [20]. Consistent with phenotypic observations, differentially expressed genes (DEGs) between diploids and autotetraploids are frequently enriched in pathways related to cell growth, cell wall organization, secondary metabolite biosynthesis, stress responses, and photosynthesis [21]. Fasano et al. hypothesizes that autopolyploidization induces a nucleotide pool imbalance, triggering a genomic shock responsible for stochastic epigenetic events in *Solanum commersonii* and *S. bulbocastanum* [22]. Further evidence from *Arabidopsis thaliana* demonstrates that autopolyploidization influences core metabolic processes, as genes involved in the TCA cycle and GABA shunt show expression trends that correlate with metabolite changes [23]. Transcriptomic analyses in *A. thaliana* further reveal that autopolyploidization primarily regulates genes associated with redox homeostasis, ABA signaling, and stress responses [24].

The jujube (*Ziziphus jujuba Mill.*), a key fruit tree native to China, encompasses rich germplasm resources with more than 1044 documented cultivars [25]. Most jujube plants are diploid under natural conditions, and only six natural triploid cultivars have been reported to date [26,27]. However, jujube breeding faces challenges such as severe embryo abortion and the difficulty of manual emasculation due to its small flowers, leading to extremely low cross-breeding efficiency [28]. As a result, polyploid breeding has become a major alternative for jujube improvement.

Jujube also possesses several inherent advantages that support its use in polyploidy research. It has abundant genetic resources, providing a solid foundation for parental selection in polyploid induction [25]. In addition, jujube has a short juvenile period and undergoes rapid flower bud differentiation [29]. Its relatively small diploid genome has been fully sequenced in the cultivar ‘Dongzao’ [30], further facilitating genetic and genomic studies. These characteristics make jujube a highly suitable model species for investigating autopolyploidization in woody plants.

We have developed a novel method for in vivo induction of homogeneous autopolyploids (IVIHA) via callus, which effectively avoids chimerism and enables rapid production of pure polyploids in Chinese jujube, sour jujube (*Z. acidojujuba* Cheng et Liu), and other woody species such as elm (*Ulmus pumila* L.) [31,32,33,34]. This approach relies on in vivo bud regeneration through callus (IVBR). Using this system, our team has successfully generated multiple autotetraploid lines. However, the phenotypic and metabolic changes induced by homoeologous duplication in the jujube genome, along with the mechanisms underlying these changes—particularly the role of phytohormones—remain poorly understood.

In this study, we conducted a systematic comparison between diploids and their respective autotetraploids of two jujube genotypes, primarily involving the morphology, cytology, metabolism, and phytohormone characteristics. The findings are expected to provide valuable insights and a practical reference for polyploid breeding in jujube.

## 2. Results

### 2.1. Ploidy Confirmation in Fruit by Flow Cytometry

**Figure 1:**
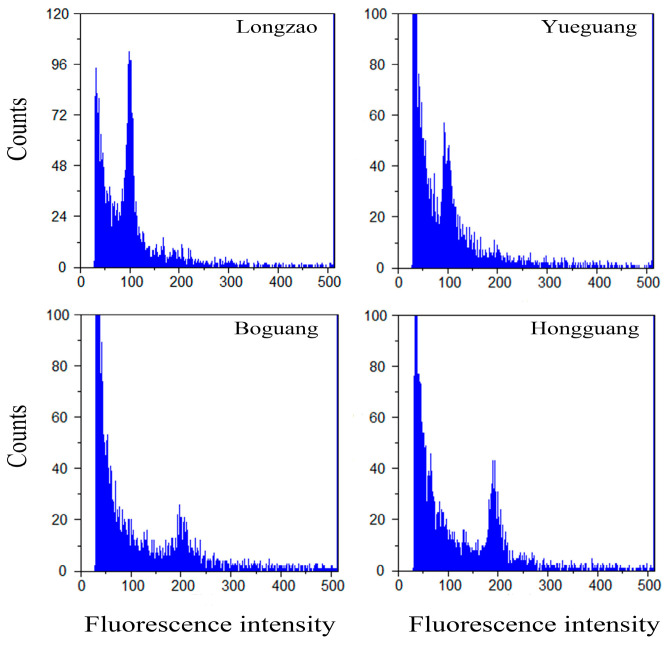
Histograms of relative DNA contents in the fruits showing ploidy levels in two pairs of jujube cultivars (‘Longzao’—2× and ‘Boguang’—4×, ‘Yueguang’—2× and ‘Hongguang’—4×).

Using an in vivo shoot regeneration system and colchicine treatment on 1–3-year-old branches of the diploid cultivars ‘Longzao’ and ‘Yueguang’, we successfully obtained the autotetraploid cultivars ‘Boguang’ and ‘Hongguang’, respectively. The resulting autotetraploid plants have been maintained by grafting and cultivated for multiple years. To confirm the genetic stability of these polyploids, we compared the DNA content of fruits from the autotetraploids and their diploid counterparts using flow cytometry. As shown in Figure 1, the DNA content peaks were approximately 100 for diploids and 200 for the induced lines, confirming their tetraploid status.

### 2.2. Morphological Changes After Autopolyploidization

#### 2.2.1. Changes in Tree Vigor

**Figure 2:**
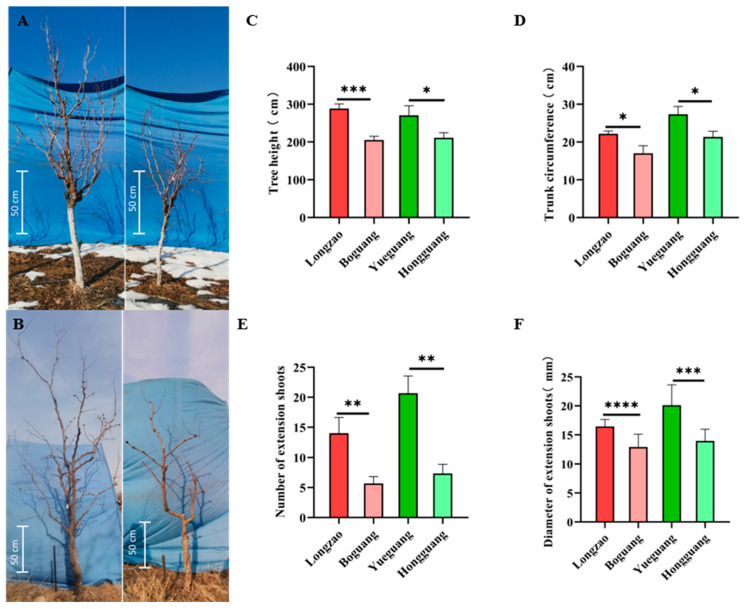
Comparison of the tree vigor between ‘Boguang’ ‘Hongguang’ and their respective diploids. (**A**) Tree growth conditions of ‘Dalilongjiao’ (left) and ‘Boguang’ (right) in the field; (**B**) Tree growth conditions of ‘Yueguang’ (left) and ‘Hongguang’ (right) in the field; (**C**) Comparison of tree height between tetraploid and diploid; (**D**) Comparison of trunk circumference between tetraploid and diploid; (**E**) Comparison of the number of extension shoots between tetraploid and diploid; (**F**) Comparison of the diameter of extension shoot between tetraploid and diploid. *, **, ***, and **** represents significant difference at *p* ≤ 0.05, *p* < 0.01, *p* < 0.001, and *p* < 0.0001 (*t*-test), respectively.

To assess the impact of autopolyploidization on tree vigor in jujube, we compared key growth traits—including tree height, trunk circumference, number of extension shoots, and shoot diameter—between the autotetraploid cultivars ‘Boguang’ and ‘Hongguang’ and their corresponding diploid progenitors, ‘Longzao’, and ‘Yueguang’. The results demonstrated a consistent reduction in growth among the tetraploids. Specifically, tree height decreased significantly by 28.9% in ‘Boguang’ and by 28.0% in ‘Hongguang’ (Figure 2A–C). Trunk circumference also declined by 23.3% and 22.0%, respectively (Figure 2D), while the number of extension shoots dropped markedly by 59.5% and 64.5% (Figure 2E). Shoot diameter was substantially reduced by 21.6% in ‘Boguang’ and 30.7% in ‘Hongguang’ (Figure 2F).

Collectively, these findings indicate that the studied autotetraploid jujubes exhibit a dwarfing phenotype characterized by a significantly reduced growth rate, fewer shoots, and overall reduced vigor.

#### 2.2.2. Changes in Bearing Shoots

**Figure 3:**
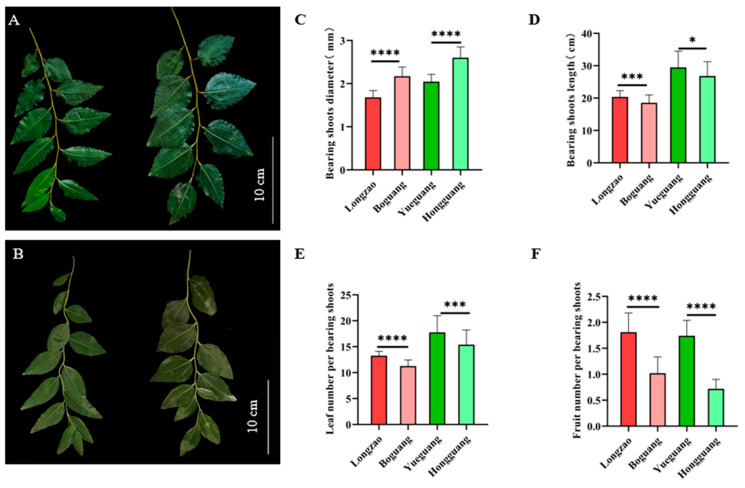
Comparison of the bearing shoots between ‘Boguang’ ‘Hongguang’ and their respective diploids. (**A**) Bearing shoots of ‘Longzao’ (left) and ‘Boguang’ (right); (**B**) Bearing shoots of ‘Yueguang’ (left) and ‘Hongguang’ (right); (**C**) Comparison of diameter of bearing shoots between tetraploid and diploid; (**D**) Comparison of length of bearing shoots between tetraploid and diploid; (**E**) Comparison of the number of leaves per bearing shoots between tetraploid and diploid; (**F**) Comparison of fruit number per bearing shoots between tetraploid and diploid. *, ***, and **** represent significant differences at *p* ≤ 0.05, *p* < 0.001, and *p* < 0.0001 (*t*-test), respectively.

To evaluate the effect of genome doubling on jujube fruiting, we examined key traits of fruiting branches (bearing shoots, Figure 3A,B). For each cultivar, 50 healthy bearing shoots were sampled to measure diameter, length, number of leaves per shoot, and fruit-setting rate (number of fruits per shoot). Compared with their diploid counterparts, the autotetraploid cultivars ‘Boguang’ and ‘Hongguang’ showed a significant increase in shoot diameter—from 1.682 mm and 2.047 mm to 2.171 mm and 2.602 mm, respectively, representing increases of 29.1% and 27.1%, both statistically highly significant (Figure 3C).

In contrast, bearing shoot length decreased by 8.9% in ‘Boguang’ and 9.2% in ‘Hongguang’, with both declines being statistically significant (Figure 3D). The number of leaves per bearing shoot also dropped significantly, by 15.1% and 13.4%, respectively (Figure 3E). Moreover, the fruit-setting rate (fruits per bearing shoot) was markedly reduced, by 43.6% in ‘Boguang’ and 58.6% in ‘Hongguang’, both with extremely significant differences (Figure 3F).

The above results demonstrate that, within each genotype, genome doubling led to shorter, thicker bearing shoots in tetraploid jujube, as well as significantly fewer leaves and a lower fruit-setting rate.

#### 2.2.3. Changes in Leaf

**Figure 4:**
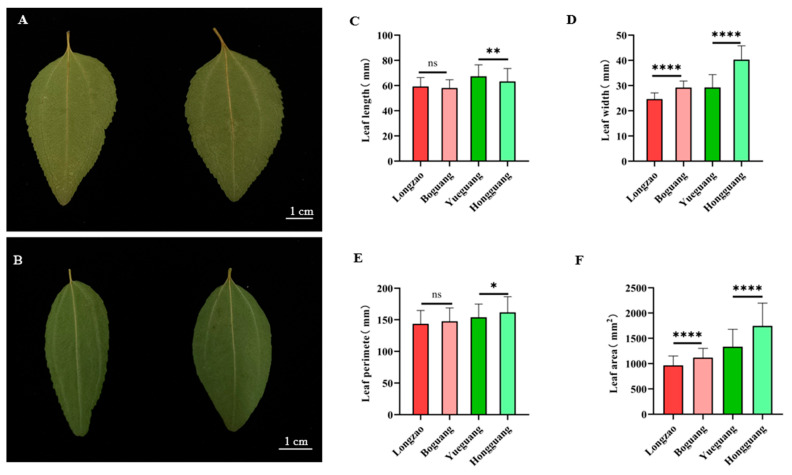
Comparison of the leaf between ‘Boguang’ ‘Hongguang’ and their respective diploids. (**A**) Leaves of ‘Longzao’ (left) and ‘Boguang’ (right); (**B**) Leaves of ‘Yueguang’ (left) and ‘Hongguang’ (right); (**C**) Comparison of leaf length between tetraploid and diploid; (**D**) Comparison of leaf width between tetraploid and diploid; (**E**) Comparison of leaf perimeter between tetraploid and diploid; (**F**) Comparison of leaf acreage between tetraploid and diploid. *, **, and **** represent significant differences at *p* ≤ 0.05, *p* < 0.01, and *p* < 0.0001 (*t*-test), respectively. ns represents no significant difference.

To assess the effect of autopolyploidization on leaf morphology in jujube, we collected 100 leaves from the middle section of bearing shoots per cultivar (Figure 4A,B) and measured leaf length, width, perimeter, and area. Comparative analysis showed that the autotetraploid ‘Boguang’ did not differ significantly from its diploid counterpart ‘Longzao’ in leaf length or perimeter (Figure 4C,E), but exhibited highly significant increases of 18.5% in leaf width and 15.9% in leaf area (Figure 4D,F). The autotetraploid ‘Hongguang’ showed a 6.0% reduction in leaf length relative to its diploid ‘Yueguang’ (Figure 4C), along with a significant increase in leaf width (37.8%), perimeter (5.1%), and area (30.8%) (Figure 4D–F), all of which were statistically significant or highly significant.

Overall, these results demonstrate that autopolyploidization generally modifies leaf traits in jujube. While ‘Boguang’ showed increased leaf width and area without changes in length or perimeter, ‘Hongguang’ displayed a slight decrease in leaf length but notable increases in width, perimeter, and area.

#### 2.2.4. Changes in Stomata

**Figure 5:**
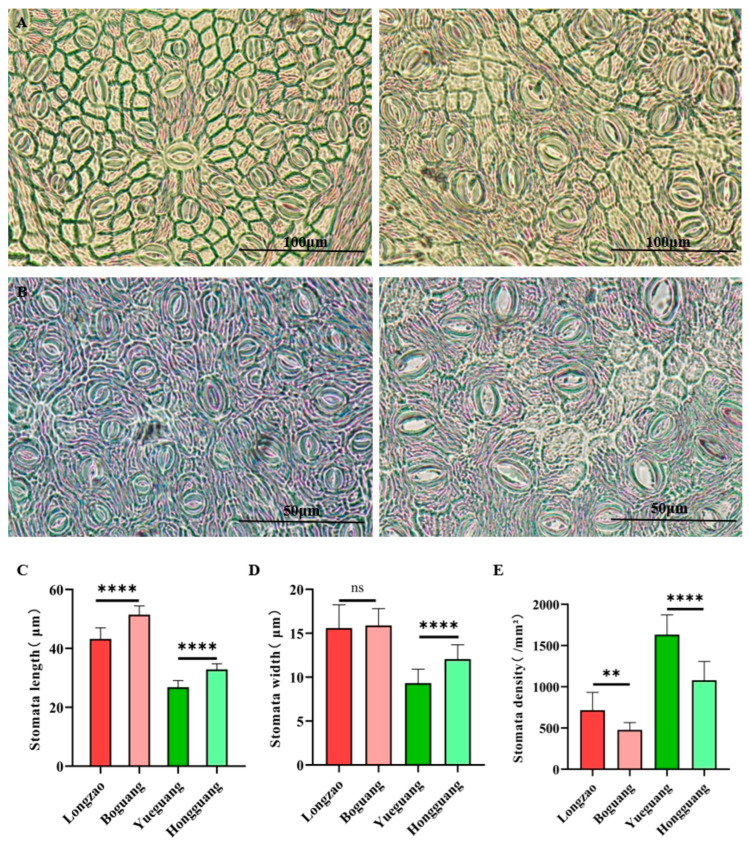
Comparison of stomatal size and density between ‘Boguang’ ‘Hongguang’ and their respective diploids. (**A**) Stomata of ‘Longjiao’ (left) and ‘Boguang’ (right); (**B**) Stomata of ‘Yueguang’ (left) and ‘Hongguang’ (right); (**C**) Comparison of stomatal length between tetraploid and diploid; (**D**) Comparison of stomatal width between tetraploid and diploid; (**E**) Comparison of stomatal density between tetraploid and diploid. ** and **** represent significant difference at *p* < 0.01 and *p* < 0.0001 (*t*-test), respectively. ns represents no significant difference.

Genome duplication is frequently associated with changes in stomatal traits. To evaluate such effects in jujube, we observed and measured the leaf stomata of the tested cultivars (Figure 5A,B). Compared with its diploid progenitor ‘Longzao’, the autotetraploid ‘Boguang’ exhibited a 19.1% increase in stomatal length (Figure 5C, *p* < 0.01), while stomatal width remained unchanged (Figure 5D). Stomatal density decreased significantly by 33.3% (Figure 5E, *p* < 0.01). In contrast, the autotetraploid ‘Hongguang’ showed increases of 22.6% in stomatal length and 29.3% in stomatal width relative to its diploid ‘Yueguang’ (Figure 5C,D, *p* < 0.01), together with a 33.8% reduction in stomatal density (Figure 5E, *p* < 0.01).

These results indicate that autopolyploidization reduced stomatal density and generally increased stomatal size within each genotype, although the extent of change in specific dimensions varied between cultivars.

#### 2.2.5. Changes in Flowers

**Figure 6:**
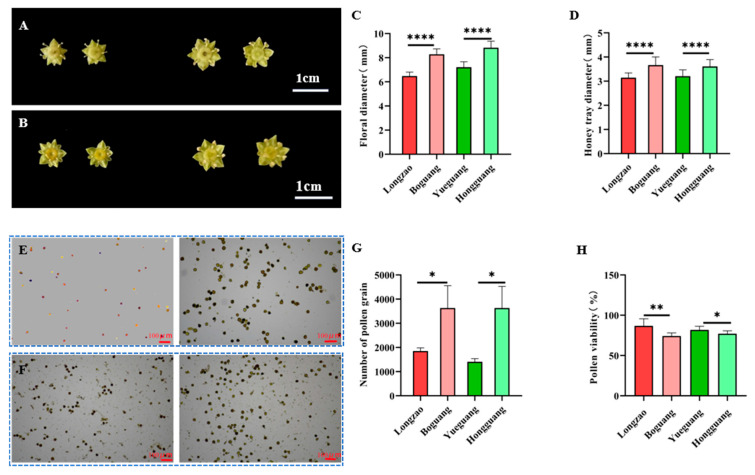
Comparison of flower characteristics between ‘Boguang’ ‘Hongguang’ and their respective diploids. (**A**) Flower of ‘Longzao’ (left) and ‘Boguang’ (right); (**B**) Flower of ‘Yueguang’ (left) and ‘Hongguang’ (right); (**C**) Comparison of flower diameter between tetraploid and diploid; (**D**) Comparison of honey tray diameter between tetraploid and diploid; (**E**) Pollen of ‘Longzao’ (left) and ‘Boguang’ (right); (**F**) Pollen of ‘Yueguang’ (left) and ‘Hongguang’ (right); (**G**) Comparison of number of pollen grain between tetraploid and diploid; (**H**) Comparison of pollen viability between tetraploid and diploid. *, **, and **** represent significant difference at *p* ≤ 0.05, *p* < 0.01, and *p* < 0.0001 (*t*-test), respectively.

To assess the impact of genome doubling on floral traits in jujube, we collected 30 flowers per cultivar at the sepal-flattening stage for measurements of flower and honey tray diameters (Figure 6A,B). Additionally, 90 anthers from yellow-bud-stage flowers were sampled for pollen counting, and 10 flowers at the sepal-flattening stage were used for pollen viability tests (Figure 6E,F).

Comparative analysis showed consistent trends across both tetraploid–diploid pairs. The autotetraploids ‘Boguang’ and ‘Hongguang’ exhibited highly significant increases in flower diameter of 27.7% and 22.4%, respectively (*p* < 0.01; Figure 6C), along with increases in honey tray diameter of 16.5% and 12.7% (*p* < 0.01; Figure 6D). Pollen production per anther also rose markedly, by 96.0% in ‘Boguang’ and 158.0% in ‘Hongguang’ (Figure 6G). In contrast, pollen viability decreased significantly in the tetraploids, by 14.5% (*p* < 0.01) and 5.7% (*p* < 0.05), respectively (Figure 6H).

These findings demonstrate that in the studied cultivars, genome doubling results in enlarged floral organs and enhanced pollen production, but compromises pollen viability. This reduction in viability may help explain the lower fruit-setting rate observed in autotetraploid jujubes.

#### 2.2.6. Changes in Fruits

**Figure 7:**
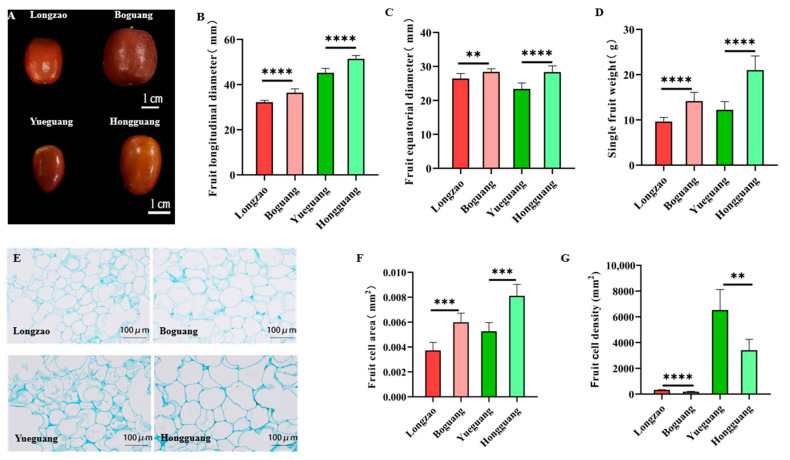
Comparison of fruit characteristics between ‘Boguang’ ‘Hongguang’ and their respective diploids. (**A**) Fruit at fully red-stage of ‘Longzao’ ‘Boguang’ ‘Yueguang’ and ‘Hongguang’; (**B**) Comparison of fruit longitudinal diameter between tetraploid and diploid; (**C**) Comparison of fruit equatorial diameter between tetraploid and diploid; (**D**) Comparison of single fruit weight between tetraploid and diploid; (**E**) Fruit cell observation of ‘Longzao’ ‘Boguang’ ‘Yueguang’ and ‘Hongguang’ at full red stage; (**F**) Comparison of fruit cell area between tetraploid and diploid fruits; (**G**) Comparison of fruit cell density between tetraploid and diploid. **, ***, and **** represent significant difference at *p* < 0.01, *p* < 0.001, and *p* < 0.0001 (*t*-test), respectively.

To evaluate the effect of genome doubling on fruit traits in jujube, we collected 30 fully red-stage fruits per cultivar (Figure 7A) and measured their longitudinal diameter, equatorial diameter, and single-fruit weight. Compared with their diploid counterparts, the autotetraploid cultivars ‘Boguang’ and ‘Hongguang’ showed significant increases in longitudinal diameter (13.0% and 11.3%), equatorial diameter (7.5% and 21.1%), and single-fruit weight (47.0% and 71.6%), respectively (Figure 7B–D).

To determine the cellular basis of fruit enlargement in tetraploids, we conducted paraffin sectioning of fully red-stage fruits. Microscopic observation revealed that the average cell area increased by 60.7% in ‘Boguang’ and 54.2% in ‘Hongguang’ (Figure 7E,F), whereas fruit cell density decreased by 42.0% and 47.7%, respectively (Figure 7E,G). All changes were statistically highly significant (*p* < 0.01). These results demonstrate that fruit enlargement in autotetraploid jujubes is primarily driven by cell expansion rather than increased cell number.

### 2.3. Comparison of Metabolite Characteristics Between Diploid and Autotetraploid Jujubes

**Figure 8:**
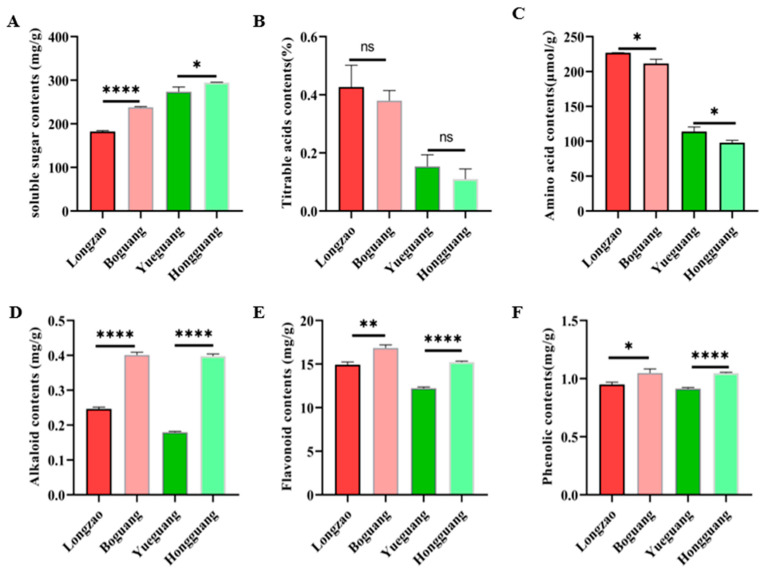
Comparison of the functional metabolites between diploid and autotetraploid jujubes. (**A**) Soluble sugar; (**B**) Titratable acid; (**C**) Amino acid; (**D**) Alkaloid; (**E**) Flavonoid; (**F**) Phenolic. *p* value according to two-sample Student’s *t* test at significance level * *p* < 0.05; ** 0.05 < *p* < 0.01; **** 0.001 < *p* < 0.0001. ns represents no significant difference.

Polyploidization has been reported to induce extensive metabolic changes in plants. To investigate whether autopolyploidization affects metabolite profiles in jujube, we quantified the contents of soluble sugars, titratable acids, amino acids, alkaloids, flavonoids, and phenolics in fruits from both diploid and tetraploid plants. As shown in Figure 8, autotetraploid fruits accumulated significantly higher levels of soluble sugars, alkaloids, flavonoids, and phenolics than their diploid counterparts. In contrast, amino acid contents were lower in autotetraploids, while titratable acid levels showed no significant difference between ploidy types.

### 2.4. Comparison of Phytohormone Levels in Leaves Between Diploid and Autotetraploid Jujubes

Phytohormones are crucial regulators of plant growth and development, influencing processes ranging from cell division and elongation to stress responses and reproductive development. While genome duplication is known to induce gigas effects and metabolic alterations, its impact on phytohormone profiles—particularly given their potent bioactivity at low concentrations—remains poorly characterized in autotetraploid jujube. To address this gap, we quantified leaf phytohormones in diploid and autotetraploid jujubes using LC-MS/MS. Differential accumulation was defined as variable importance in projection (VIP) ≥ 1 and fold change ≥ 1.2 or ≤0.83.

**Figure 9:**
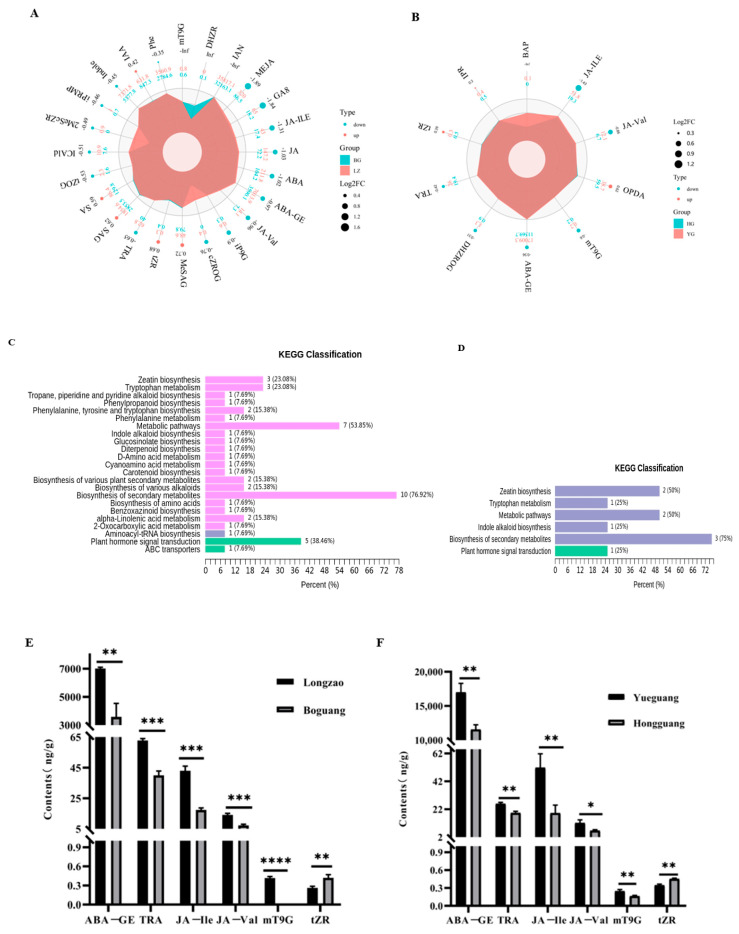
Comparison of phytohormone levels in leaves between diploid and autotetraploid jujubes. (**A**) Differentially accumulated phytohormones between ‘Longzao’ and ‘Boguang’ jujubes; (**B**) Differentially accumulated phytohormones between ‘Yueguang’ and ‘Hongguang’ jujube. Percent (%): the ratio between the number of differentially expressed metabolites mapped to a particular metabolic pathway and the total number of KEGG-annotated differential metabolites detected in the experiment. The same below. (**C**) KEGG enrichment analysis of differentially accumulated phytohormones between ‘Longzao’ and ‘Boguang’ jujubes; the pink bars represent metabolism, the blue-violet bar represents genetic information processing, and the green bars represent environmental information processing. (**D**) KEGG enrichment analysis of differentially accumulated phytohormones between ‘Yueguang’ and ‘Hongguang’ jujubes; the blue-violet bars represent metabolism, and the green bars represent environmental information processing. (**E**) The contents of 6 differentially accumulated phytohormones in ‘Longzao’ and ‘Boguang’ jujubes; (**F**) The contents of 6 differentially accumulated phytohormones in ‘Yueguang’ and ‘Hongguang’ jujubes.

A total of 24 and 10 phytohormones were differentially accumulated in ‘Boguang’ and ‘Hongguang’ tetraploids compared to their diploid counterparts, respectively (Figure 9A,B). Specifically, ‘Boguang’ showed lower levels of GA_8_ and MEJA, while ‘Hongguang’ exhibited reduced JA-Ile and JA-Val. KEGG enrichment analysis indicated that these hormones were primarily associated with secondary metabolite biosynthesis and general metabolic pathways (Figure 9C,D). Notably, six phytohormones—ABA-GE, TRA, JA-Ile, JA-Val, mT9G, and tZR—were consistently differentially accumulated in both cultivars. Among them, tZR was upregulated in tetraploids, while the other five were downregulated (Figure 9E,F). All six phytohormones exhibited consistent alteration patterns across the two comparison groups.

## 3. Discussion

### 3.1. Common Phenotypic Variations Induced by Autopolyploidy and Their Implications for Jujube Cultivation

Autopolyploidization serves as an important breeding strategy, though the phenotypic consequences of genome doubling remain poorly characterized across plant species. In this study on jujube, we identified multiple common variations in autopolyploids with different genetic backgrounds, encompassing both morphological and metabolic traits (Supplementary Table S1). For instance, autopolyploid jujubes displayed organ enlargement, including larger flowers, leaves, and fruits. Similar gigas effects have been reported in other species: autotetraploid carmine radish (*Raphunas sativus* L.) developed larger leaves, longer and heavier taproots, and enlarged floral organs [35], while autotetraploid mulberry (*Morus alba* L.) showed increased leaf and fruit sizes [9]. Although genome doubling generally promotes organ enlargement in tetraploid jujube, the specific expression of these traits is genotype-dependent. For example, compared to their diploid progenitors, the autotetraploid ‘Boguang’ exhibited increases only in leaf width and area, whereas ‘Hongguang’ displayed broader improvements in overall leaf morphology, despite a slight reduction in leaf length.

In addition to these beneficial agronomic traits, autopolyploidization also induced certain undesirable effects in jujube, such as reduced shoot number, lower pollen viability, and decreased fruit set per bearing shoot. Moreover, autotetraploid jujube trees exhibited dwarfism relative to diploids, a phenomenon consistent with observations in apple [12] and garlic [13] autotetraploids.

We found that although autotetraploid jujube produced more pollen grains, their viability was significantly lower, resulting in reduced fruit setting. This suggests that the increased pollen quantity does not compensate for the decline in fertility, ultimately leading to impaired reproductive performance in autopolyploid jujube. While reduced pollen viability may facilitate seedless fruit formation, impaired pollination and fertilization generally lead to lower fruit and seed production.

Although autotetraploid jujube shows weakened growth, dwarfism, and lower fruit set traits that may reduce individual tree productivity, dwarfism is a key attribute in modern high-density jujube orchards. The use of dwarfing rootstocks to control tree vigor allows for intensified planting systems that significantly increase yield per unit area. Therefore, autotetraploid jujube represents a valuable dwarfing germplasm resource with promising practical applications in breeding programs.

### 3.2. Mechanisms and Significance of Enhanced Secondary Metabolites in Autopolyploids

Compared to diploid jujube, autotetraploids exhibited reduced amino acid content but elevated levels of flavonoids, alkaloids, phenolics, and soluble sugars. Similar metabolite shifts have been observed in other polyploid plant species. For instance, tetraploid *Catharanthus roseus* showed increased terpenoid indole alkaloid content [36], autotetraploid *Cichorium intybus* accumulated 1.9-fold higher leaf phenolics with a 10-fold increase in chlorogenic acid [37], and tetraploid *Bletilla striata* contained more chlorophyll, crude polysaccharides, and total phenols in leaves [38]. In lemon balm (*Melissa officinalis* L.), total flavonoid content rose with increasing colchicine concentration up to 0.1% [39]. These consistent observations across diverse species suggest that polyploidization triggers a fundamental reprogramming of metabolic networks, which can be attributed to several interconnected mechanisms.

The enhancement of secondary metabolites in autopolyploids arises from a complex interplay of genomic, transcriptional, and metabolic adaptations. These include gene dosage effects, transcriptional reprogramming, resource reallocation, and improved ecological adaptability. A key genomic underpinning is the alteration of chromatin architecture. For instance, Zhou et al. reported that autopolyploid rice contains more accessible chromatin regions (ACRs) in euchromatin, which was correlated with higher leaf flavone levels. They found elevated ACR density and expression of a key phenylpropanoid pathway gene, cinnamic acid 4-hydroxylase (C4H), in tetraploids [11]. Such transcriptional reprogramming is further driven by specific transcription factors. In industrial hemp autotetraploids, Tang et al. identified several bHLH and MYB proteins, along with key catalytic genes like flavonol synthase, as critical regulators of phenylpropanoid metabolism [40]. The concomitant phenomenon of metabolic network redundancy—where gene duplication provides multiple isoforms of enzymes, increasing pathway flexibility and robustness—may further stabilize these metabolic shifts. These genomic and transcriptional changes are supported by a reshaped primary metabolism that provides essential precursors and energy. Meng et al. found that tetraploid rice exhibits altered carbohydrate metabolism, including enhanced starch turnover and monosaccharide phosphorylation, which facilitates resource redistribution. Moreover, stronger glycolytic and TCA cycle activity at night in tetraploids supports more efficient energy production [41]. In essence, the polyploid genome acts as a master regulator, inducing transcriptional networks that both directly upregulate secondary metabolic pathways and reconfigure central carbon metabolism to fuel them. From an ecological perspective, these metabolic enhancements confer a competitive advantage. Milosavljevic et al. demonstrated that even minor metabolic advantages can facilitate the invasion of polyploids into diploid populations through nutrient competition [42]. The elevated defense compounds like alkaloids and phenolics likely constitute a key component of this advantage, by inhibiting competitors or herbivores.

The accumulation of these secondary metabolites significantly enhances the agronomic and pharmacological value of autopolyploid jujube. In plants, flavonoids, alkaloids, and phenolics collectively contribute to improved stress resilience, UV protection, and defense against pathogens [43,44,45,46,47]. This translates directly into agronomic benefits such as improved field survival and postharvest quality. From a pharmacological standpoint, this metabolic profile is highly desirable. The synergistic action of these compounds underpins a broad spectrum of bioactivities relevant to human health. They are widely recognized for their potent anti-inflammatory, antioxidant, antimicrobial, and anticancer properties [48,49,50,51,52,53,54,55,56,57,58,59,60,61,62,63,64], positioning autopolyploid jujube as a promising resource for developing functional foods or plant-derived pharmaceuticals.

Future research integrating multi-omics data across different plant species and ploidy levels will be crucial to disentangle the primary drivers from the secondary consequences in this complex regulatory network, and to harness these mechanisms for targeted crop improvement.

### 3.3. Reconstruction of Endogenous Hormone Homeostasis and Its Phenotypic Implications in Autotetraploid Jujube

Phytohormones are pivotal regulators of plant growth, development, and stress responses. Our study revealed a consistent reconstruction of hormonal homeostasis in both autotetraploid–diploid cultivar pairs, characterized by a significant reduction in several hormones—ABA-GE, TRA, JA-Ile, and JA-Val—coupled with an elevation of the cytokinin tZR. This shifted hormonal landscape likely underlies the distinct phenotypic alterations in autotetraploid jujube.

The coordinated decline in jasmonates and auxin-related compounds may collectively contribute to the observed dwarfism. Jasmonates (JAs), such as JA-Ile and JA-Val, are well-known growth inhibitors that repress plant growth to prioritize defense responses [65]. The reduced levels of these JAs in our autotetraploids are consistent with a release from growth inhibition, yet the overall dwarfism suggests a more complex regulation. This could be explained by the concurrent decrease in tryptamine (TRA), an auxin-related compound essential for directing carbon and nitrogen flux into growth-promoting pathways [66]. Thus, the dwarfism is likely not due to a single hormone but to the dual suppression of both jasmonate and auxin-mediated growth signals.

Conversely, the elevated cytokinin tZR may drive the gigas effect in specific organs. tZR, a highly active cytokinin, promotes cell division and organogenesis [67,68]. Its increased level in autotetraploids provides a plausible mechanism for the enlargement of certain organs (the gigas effect), a common polyploidy trait. Critically, the antagonistic interaction between cytokinins and auxin [69,70] is exemplified here: the reduction in TRA (auxin pathway) may have perturbed the hormonal balance, creating a favorable context for cytokinin-driven growth to manifest in specific tissues.

The implications of altered ABA and JA homeostasis extend beyond growth to stress adaptation and secondary metabolism. The consistent decrease in the ABA storage form, ABA-GE, suggests an altered ABA turnover dynamics [71,72], which could modulate the plant’s stress response strategy. Furthermore, the downregulation of JA signaling may have a dual impact: while potentially compromising immediate insect resistance by reducing glandular trichome formation [73], it might also reallocate resources from JA-mediated defense towards the enhanced biosynthesis of constitutive defense compounds, such as the flavonoids and alkaloids documented in Section 3.2. This potential trade-off between inducible (JA) and constitutive chemical defense warrants further investigation.

In summary, autopolyploidy in jujube does not simply suppress hormone levels but orchestrates a comprehensive reprogramming of the hormonal network. The core phenotype—dwarfism coupled with organ gigas—can be interpreted as the result of this new homeostasis: dampened JA and auxin-related growth versus enhanced cytokinin-driven development. This hormonal shift is likely intertwined with the rewiring of secondary metabolism and represents a fundamental adaptation to the polyploid state. Future research employing transcriptional analysis and exogenous hormone application is crucial to validate these interactions and establish causal relationships.

Our study also has certain limitations. The two cultivar pairs selected for comparison—‘Longzao’ and ‘Boguang’, as well as ‘Yueguang’ and ‘Hongguang’—were cultivated in different locations (Fuping and Zanhuang, respectively) and grafted onto different rootstocks (‘Pozao’ and ‘Zanhuangdazao’, respectively). Although both sites are located within Hebei Province and share broadly similar macroclimatic conditions, differences in soil composition, microclimate, and notably, rootstock physiological characteristics, may significantly influence morphological and metabolic traits. Therefore, while comparisons between diploid and autotetraploid plants within each cultivar pair are valid and reliable due to their shared environment and rootstock, direct comparisons of the extent of ploidy effects between the two pairs should be interpreted with caution. The observed phenotypic differences cannot be attributed solely to ploidy level but are also influenced by genotype-by-environment and genotype-by-rootstock interactions. In conclusion, the findings of this study regarding ploidy effects are applicable only within specific genetic backgrounds, and extrapolation to other genotypes requires further validation under controlled conditions.

## 4. Material and Methods

### 4.1. Plant Materials

We employed two autotetraploid jujube cultivars, ‘Hongguang’ and ‘Boguang’, together with their diploid counterparts ‘Yueguang’ and ‘Longzao’. Among them, ‘Hongguang’ and ‘Boguang’ have been officially certified (HB-SV-ZJ-011-2017 and 20230756, respectively) by provincial and national authorities [32,35], and ‘Longzao’ was sourced from Dali County, Shanxi Province.

### 4.2. Growth Conditions

All cultivars were top-grafted onto mature jujube rootstocks and managed under standard horticultural practices. ‘Longzao’ and its autotetraploid ‘Boguang’ were top grafted on ‘Pozao’ jujube plants at Dianfang, Fuping County, Hebei Province in 2018. ‘Yueguang’ and its autotetraploid ‘Hongguang’ were top grafted on ‘Zanhuangdazao’ at Haojiazhuang, Zanhuang County, Hebei Province in 2017. The region has a continental climate, with mean annual temperatures of 12.6~13.3 °C and annual precipitation of 812.3~975.3 mm. Phenotypic data were collected during the 2021 growing season.

### 4.3. Morphological Analysis

Morphological traits were assessed at key developmental stages. Following leaf fall, tree height, trunk circumference, number of extension shoots, and the diameter of both extension and bearing shoots were recorded. At harvest, bearing shoot diameter, fruit length and diameter, and single fruit weight were measured. During full bloom, floral diameter, honey tray diameter, pollen viability, and pollen grain number were evaluated. The number of fruits per bearing shoot was recorded in late July, after physiological fruit drop.

For each cultivar, three uniformly growing, healthy trees were selected for measurement. The total number of extension shoots per tree was counted and averaged. Shoot diameter was determined by randomly selecting and measuring 10~15 extension shoots per tree.

### 4.4. Cytological Observation of Stomata, Pollen and Fruit Pulp Cell

Cytological examinations were performed following a published protocol [74] with modifications. For stomatal analysis, ten healthy leaves per cultivar were randomly sampled from multiple trees. Abaxial epidermal impressions were obtained using transparent nail polish and adhesive tape, mounted on glass slides, and covered with coverslips. Stomatal length, width, and density were measured at 10× magnification using a Nikon Y-T55 microscope (Nikon Corporation, Tokyo, Japan). Stomatal characteristics were quantified with NIS Elements D 5.41.00 software, and three fields analyzed per leaf.

Pollen number was quantified by collecting 30 healthy bearing shoots at the middle bud-yellowing stage. A total of 90 plump, non-dehiscent anthers were excised and equally distributed into three vials. After natural dehiscence, pollen was suspended in 2 mL of 1% sodium hexametaphosphate per vial. Grains were counted using a hemocytometer under low magnification (with a 40× eyepiece), and the number per anther (N) was calculated as: N = (Total count in 400 squares × 10,000 × 2)/30. Three replicates were performed per vial.

Pollen viability was assessed using ten newly opened flowers per cultivar at the sepal-flattening stage. One anther per flower was placed on a labeled slide, macerated in distilled water, stained with I_2_-KI, and covered. Samples were observed under a Nikon Y-T55 microscope (Nikon Corporation, Tokyo, Japan) at low magnification (with a 10× eyepiece), with pollen grains scored as viable (brown to darkly stained) or non-viable (unstained or lightly stained). The percentage of viable pollen was calculated per slide, with three replicates per sample.

Three mature fruits per cultivar were randomly selected for pulp cell examination. From each fruit, two flesh segments were excised from opposite sides and fixed in 4% paraformaldehyde. The samples subsequently underwent standard histological processing including trimming, dehydration, embedding, sectioning, staining, and mounting according to established laboratory protocols. Section observation was performed using an Eclipse Ci-L microscope (Nikon Corporation, Tokyo, Japan) at 200× magnification. Images were captured under consistent illumination conditions with tissue sections positioned to fill the field of view. Pulp cell number and area were quantified using Image-Pro Plus 6.0 software, with measurements calibrated to millimeter units. The average pulp cell area was calculated as total cell area divided by cell count per image.

### 4.5. Extraction and Analysis of Metabolites

Metabolite contents were determined using commercial assay kits or standard biochemical methods as outlined below.

Amino acids were quantified with a commercial kit (Solarbio, Beijing, China) [75]. Briefly, 0.1 g of tissue was homogenized in 1 mL of Reagent 1, transferred to a 1.5 mL tube, and extracted in a boiling water bath for 15 min. After cooling and centrifugation (10,000 rpm, 4 °C, 10 min), the supernatant was collected for analysis according to the manufacturer’s instructions.

Alkaloids were measured using an assay kit (Comin, Suzhou, China) [76]. Approximately 0.1 g of sample was mixed with 0.1 mL of Reagent I and 0.9 mL of 80% ethanol, followed by ultrasonic extraction for 60 min. After centrifugation (8000 rpm, 25 °C, 10 min), the supernatant was analyzed. A blank was prepared by mixing 0.1 mL Reagent I with 0.9 mL of 80% ethanol.

Soluble sugars were analyzed with a kit (Comin, Suzhou, China) [77]. A total of 0.1 g of fresh fruit pulp was homogenized in 1 mL of distilled water and incubated in a 95 °C water bath for 10 min. After cooling and centrifugation (8000 rpm, 25 °C, 10 min), 0.1 mL of supernatant was diluted with 0.9 mL distilled water for subsequent testing as per the kit protocol.

Titratable acidity was determined by NaOH-phenolphthalein titration and expressed as malic acid equivalent (conversion factor 0.067) [78]. The content was calculated as: C = (V/m) × (V_2_ × C_s_)/V_1_ × 0.067 × 100%. Where C is titratable acid (%), V is total extract volume (mL), V_1_ is sample aliquot volume (mL), V_2_ is NaOH volume consumed (mL), C_s_ is NaOH concentration (mol/L), and m stands for sample mass (g). Analyses were performed in triplicate.

Total flavonoids were measured by colorimetry [79]. A 5 mL aliquot of sample or rutin standard (0~100 mg/L) was reacted with 0.6 mL of 5% NaNO_2_ for 6 min, followed by 0.4 mL of 10% Al(NO_3_)_3_ for 6 min and 2 mL of 4% NaOH. The volume was adjusted to 10 mL with ethanol, and absorbance was read at 511 nm. Results are expressed as mg rutin equivalent per g fresh weight (mg RE/g FW).

Total phenolics were determined using the Folin–Ciocalteu method [80]. A 0.2 mL aliquot of sample or gallic acid standard (0~10 mg/L) was mixed with 0.5 mL Folin-phenol reagent and 1.5 mL of 7.5% Na_2_CO_3_, incubated in the dark for 2 h, and absorbance was measured at 765 nm. Results are expressed as mg gallic acid equivalent per g fresh weight (mg GAE/g FW).

### 4.6. Determination of Endogenous Phytohormone Levels with LC-MS/MS

To determine the levels of different phytohormones in both cultivars, the young leaves, approximately 3~4 cm length, were collected for use, The samples were collected immediately and frozen in liquid nitrogen and stored at −80 °C to use. Phytohormones contents were detected by MetWare (http://www.metware.cn/, accessed on 15 November 2023) based on the AB Sciex QTRAP 6500 LC-MS/MS platform. Briefly, the frozen samples were dissolved in 1 mL methanol/water/formic acid (15:4:1, *v*/*v*/*v*). And then add 10 μL internal standard mixed solution (100 ng/mL) into the extract as internal standards (IS) for the quantification. The mixture was vortexed for 10 min, then centrifuged for 5 min (12,000 rpm, at 4 °C), and the supernatant was transferred to clean plastic microtubes, followed by evaporation to dryness and dissolved in 100 μL 80% methanol (*v*/*v*), and filtered through a 0.22 μm membrane filter for further LC-MS/MS analysis. The endogenous contents of cytokinins (DHZR, IPR, mT9G and BAP), auxin (ICAld, MEIAA, ILA, IAA, IAA-Asp, Indole, TRA, and IAN), ACC, Gibberellins (GA_3_, GA_8_ and GA_34_), salicylic acid (SA and MeSAG), Abscisic acid (ABA and ABA-GE),5DS and jasmonates (JA, MeJA, JA-Ile and OPDA) were then measured under the following conditions: Liquid condition: column, Waters ACQUITY UPLC HSS T3 C18 (100 mm × 2.1 mm i.d.,1.8 µm); solvent system, water with 0.04% acetic acid (A), acetonitrile with 0.04% acetic acid (B); gradient program, started at 5% B (0~1 min), increased to 95% B (1~8 min), 95% B (8~9 min), finally ramped back to 5% B (9.1~12 min); flow rate, 0.35 mL/min; temperature, 40 °C; injection volume: 2 µL. ESI-MS/MS conditions: Linear ion trap (LIT) and triple quadrupole (QQQ) scans were acquired on a triple quadrupole-linear ion trap mass spectrometer (QTRAP), QTRAP^®^ 6500+ LC-MS/MS System, equipped with an ESI Turbo Ion-Spray interface, operating in both positive and negative ion mode and controlled by Analyst 1.6.3 software (Sciex, Framingham, MA, USA). The ESI source operation parameters were as follows: ion source, ESI+/−; source temperature 550 °C; ion spray voltage (IS) 5500 V (Positive), −4500 V (Negative); curtain gas (CUR) was set at 35 psi, respectively. Phytohormones were analyzed using scheduled multiple reaction monitoring (MRM). Data acquisitions were performed using Analyst 1.6.3 software (Sciex). Multiquant 3.0.3 software (Sciex) was used to quantify all metabolites. Mass spectrometer parameters including the declustering potentials (DP) and collision energies (CE) for individual MRM transitions were performed with further DP and CE optimization. A specific set of MRM transitions were monitored for each period according to the metabolites eluted within this period. All standard compounds were purchased from Sigma Aldrich. Three replicates were conducted per sample per treatment.

### 4.7. Data Analysis

To provide better quality of pictures, the unrelated edges of several photos taken from the field were cropped using photoshop without distorting the image. Statistical analyses, including normality/homoscedasticity checks and Student’s *t*-test as well as the generation of bar graphs, were conducted using GraphPad Prism software (version 8.0.2). The differences between diploid (control) and autotetraploid plants were considered significant at *p* < 0.05, *p* < 0.01, *p* < 0.001, *p* < 0.0001 levels. Differential accumulated phytohormone and KEGG analysis was performed using the Metware Cloud, a free online platform for data analysis (https://cloud.metware.cn, accessed on 15 November 2023).

## 5. Conclusions

This study systematically investigated the effects of autopolyploidization on the phenotype, metabolome, and phytohormones of jujube using two genetically distinct pairs of diploids and their corresponding autotetraploid materials. The results revealed that autotetraploid jujube plants exhibited a combination of characteristics, including organ gigantism, increased secondary metabolite content, reduced pollen viability, and plant dwarfism. Furthermore, phytohormone analysis showed elevated levels of cytokinin tZR in the tetraploid materials, while the levels of ABA-GE, TRA, JA-Ile, JA-Val, and mT9G were generally decreased. These findings suggest that the interaction and synergistic effects among phytohormones may serve as the internal driver underlying the phenotypic changes in tetraploid jujube.

## Data Availability

All data generated or analyzed during this study are included in this published article. Further inquiries can be addressed to the corresponding author.

## Supplementary Materials

The following are available online at https://www.mdpi.com/article/10.3390/plants14233588/s1, Table S1: Comparison of morphological characteristics, metabolic contents and phytohormone levels between diploid and autotetraploid jujubes.
