# Supplementary material for: Phenotypic and Metabolic Variations Induced by Autopolyploidization in Chinese Jujube Cultivars
Source: Plants (Basel). 2025 Nov 25;14(23):3588. doi: 10.3390/plants14233588 (PMC12694531; doi:10.3390/plants14233588)
Supplement: Supplementary file 1 [file plants-14-03588-s001.zip › plants-3900285-supplementary.pdf]

Table S1. Comparison of morphological characteristics, metabolic contents and phytohormone levels between diploid and autotetraploid jujubes

**Morphological characteristics**	**Longzao**	**Boguang**	**Yueguang**	**Hongguang**
Height of tree (cm)	288.3±12.58	205.0±10.00***	270.3±25.66	211.0±13.53*
Trunk circumference(cm)	22.17±0.7638	17.00±2.000*	27.33±2.082	21.33±1.528*
Numbers of extension shoots	14.0±2.646	5.7±1.16*	20.7±2.9	7.3±1.5*
Diameter of extension shoots (mm)	16.45±1.198	12.90±2.226****	20.12±3.517	13.95±2.032***
Diameter of bearing shoots (mm)	1.682±0.1563	2.171±0.2138****	2.047±0.1674	2.602±0.2492****
Bearing shoots length(cm)	20.36±1.964	18.54±2.452***	29.50±5.009	26.80±4.460*
Leaf number per bearing shoots	13.25±0.8397	11.25±1.193****	17.75±3.209	15.38±2.844***
Fruit number per bearing shoots	1.810±0.3695	1.020±0.3120****	1.740±0.2989	0.7200±0.1814****
Leaf length (mm)	59.32±7.068	58.08±6.530^{ns}	67.37±9.102	63.32±10.19**
Leaf width (mm)	24.64±2.455	29.21±2.600****	29.26±5.079	40.31±5.465****
Leaf perimete(mm)	143.6±21.19	147.6±21.19^{ns}	153.9±21.03	161.8±24.79*
Leaf area (mm^2)	965.2±185.3	1119.0±182.7****	1334.0±344.6	1745.0±452.7****
Stomata length (μm)	43.24±3.718	51.48±2.946****	26.81±2.264	32.88±1.884****
Stomata width (μm)	15.58±2.657	15.88±1.923^{ns}	9.33±1.585	12.06±1.630****
Stomata density (mm^2)	716±217.0	477±89.2**	1632±240.9	1080±227.6****
Floral diameter (mm)	6.480±0.3221	8.276±0.4508****	7.208±0.4505	8.826±0.5470****
Honey tray diameter (mm)	3.144±0.1959	3.664±0.3381****	3.205±0.2640	3.611±3.611****
Number of pollen grain	1852±128.3	3630±925.2*	1407±128.3	3630±898.1*
Pollen viability (%)	86.76±8.800	74.16±3.960**	81.75±4.511	77.11±3.580*
Fruit longitudinal diameter (mm)	32.25±0.7548	36.44±1.699****	45.23±1.960	51.43±1.420****

Fruit equatorial diameter (mm)	26.45±1.466	28.43±0.8936**	23.40±1.757	28.34±1.853****
Weight of single fruit (g)	9.63±0.9003	14.16±1.939****	12.24±1.824	21.01±3.136****
Cell area of fruit (mm^2)	0.003727±0.0006413	0.005990±0.0007285***	0.005260±0.0006984	0.008113±0.0009201***
Fruit cell density (mm^2)	325.9±16.56	188.9±22.34****	6526±1594	3415±839.8**
**Metabolic contents**	**Longzao**	**Boguang**	**Yueguang**	**Hongguang**
soluble sugar contents (mg/g)	182.5±2.026	237.8±1.787****	273.6±11.02	294.8±0.8218*
Titrable acids contents(%)	0.4267±0.07506	0.3800±0.03464	0.1533±0.04041	0.1100±0.03464
Amino acid contents(μmol/g)	226.9±0.09920	211.6±5.903^{ns}	114.1±6.300	98.05±3.324^{ns}
Alkaloid contents (mg/g)	0.2463±0.005041	0.4012±0.007583****	0.1793±0.002542	0.3979±0.005977****
Flavonoid contents (mg/g)	14.92±0.3244	16.85±0.3579**	12.23±0.1464	15.23±0.1190****
Phenolic contents(mg/g)	0.9490±0.02023	1.048±0.03565*	0.9128±0.01062	1.047±0.007529****
**Phytohormone levels**	**Longzao**	**Boguang**	**Yueguang**	**Hongguang**
ABA-GE(ng/g)	7013.93866666667±92.5251420	3590.1070000 ± 955.6998932**	17009.2833333 ± 1300.0141129	11569.6966667 ± 680.4539075**
TRA(ng/g)	7013.9386667± 1.2432603	39.9580133 ± 2.8533975***	25.9673867±0.9186998	19.4070233±1.0840255**
JA-ILE(ng/g)	62.7720533 ±3.0218917	17.3657133 ± 1.3495245***	51.7950133±9.9181844	19.2546767±5.7583747**
JA-Val(ng/g)	42.9814833 ±0.9941858	7.2805770±0.7462429***	12.1333093±2.2375618	6.6789590± 0.6821305*
mT9G(ng/g)	14.1482900 ±0.0267221	0.0000000 ± 0.0000000****	0.2471163±0.0269743	0.1627887±0.0122258**
tZR(ng/g)	0.4150163 ±0.0249189	0.4191893±0.504028**	0.3459147±0.0186834	0.4517333±0.0126141**

Note: Data are shown as mean ± standard deviation. *P* value according to two-sample Student's t test at significance level **P* < 0.05; ***P* < 0.01， ****P* < 0.001， *****P* < 0.0001, ns stands for non-significant
